# A novel reverse transcription recombinase polymerase amplification assay for rapid detection of GI.1 genotype of rabbit hemorrhagic disease virus

**DOI:** 10.3389/fvets.2023.1056601

**Published:** 2023-03-10

**Authors:** Lianzhi Zhang, Qiaoya Zhao, Ye Tian, Yi Tang, Yixin Wang, Bing Huang

**Affiliations:** ^1^College of Animal Science and Veterinary Medicine, Shandong Agricultural University, Tai'an, China; ^2^Institute of Poultry Sciences, Shandong Academy of Agricultural Sciences, Jinan, China

**Keywords:** rabbit hemorrhagic disease virus, GI.1 genotype, isothermal, recombinase polymerase amplification, rapid detection method, epidemiological surveillance

## Abstract

Rabbit Viral Hemorrhagic Disease (RHD) is a highly contagious and fatal infection, resulting in considerable economic losses to the rabbit industry. Consequently, it is essential to develop a fast and accurate diagnostic method for RHDV GI.1. In this study, a rapid simple reverse transcriptase recombinase polymerase amplification (RTRPA) for RHDV GI.1 was successfully developed using specific primers to RHDV GI.1 VP60 gene. Results indicated that the entire amplification process could be achieved in an isothermal condition at 40°C for 30 minutes, with good specificity and no reaction to other common rabbit disease pathogens, and a high sensitivity of upto 0.1LD_50_ of RHDV GI.1. Then, RT-RPA method was used to detect 1144 clinical samples, and the positive rates were 0.95%, 1.29% and 2.50% in Zaozhuang, Linyi, and Liaocheng in Shandong Province, respectively (the Fisher's exact test, *P* = 0.413), suggesting that there is no significant difference in RHDV GI.1 infection among the different regions. In conclusion, this study established a RT-RPA assay which is suitable for quick detection and monitoring of RHDV GI.1, thus making it a viable option for epidemiological surveillance.

## 1. Introduction

Rabbit hemorrhagic disease (RHD) is an acute, septic, highly contagious and fatal disease caused by rabbit hemorrhagic virus (RHDV) infection (1). The disease first broke out in Wuxi, Jiangsu Province, China in 1984 and then spread to many regions around the world, which attracted the attention of scholars all over the world. The main clinical symptoms were neurological symptoms, respiratory symptoms, slow response and anorexia. The disease is characterized by rapid transmission and high mortality. Infected rabbit generally dies within 1–2 days after fever, and the mortality is as high as 70%-90%. The virus seriously damaged the ecological environment of rabbits, endangered rare hare species and affected the development of rabbit industry. In 2010, an additional RHDV, phylogenetically, and antigenically distinct from RHDV, emerged in Europe and was called RHDV2. A new RHDV isolate collected from infected rabbits at farms in the Sichuan province of China in April 2020, representing the first report of RHDV2 in China (2). According to the new genome classification, the genomes of classical RHDV are classified as GI.1, and the new emerged RHDV2 is now classified as GI.2. At present, RHD caused by RHDV GI.1 is highly epidemic in Chinese rabbit farms, even though some rabbits have been vaccinated with commercial vaccines, they still remain susceptible to the virus. Therefore, it is essential to create a rapid detection method for RHDV GI.1.

Since RHD can exist stably in the environment and transmit quickly, therefore, it is vital for RHDV GI.1 rapid detection in rabbit flocks. Various methods can be used to detect the virus, such as hemagglutination assay (HA), enzyme-linked immunosorbent assay (ELISA), reverse-transcription PCR (RT-PCR) (3) and quantitative real-time RT-PCR (qRT-PCR). However, these methods all have shortcomings such as time-consuming and low sensitivity. In addition, some methods are very demanding on instruments and equipment. Therefore, the development of a portable rapid test to monitor RHDV GI.1 infection is crucial in field diagnosis.

Recombinase polymerase amplification (RPA) is known as a novel nucleic acid detection technology that has several advantages compared with conventional PCR assay. RPA technology mainly relies on three enzymes: recombinase that can bind single stranded nucleic acid (oligonucleotide primer), single stranded DNA binding protein (SSB) and strand replacement DNA polymerase. Under isothermal conditions (generally constant at 37–39°C), the reaction process is very fast, amplification products usually can be detected within 20–40 min, then the amplified fragments can be observed by agarose gel electrophoresis (4). To our knowledge, there was no report on the RT-RPA method for the rapid detection of RHDV GI.1.

The RHDV GI.1 genome is a single-stranded, positive-strand RNA which composed of 7,437 nucleotides (nt), including two open-reading frames (ORFs): positive sense ORF1 and ORF2. ORF1 encodes a large polyprotein which then hydrolyzed to some non-structural proteins and the capsid protein VP60. ORF2 encodes the minor structural protein VP10 (5–8). VP60 protein is not only the capsid protein of the virus, but also the only structural protein of the virus (9, 10). In this study, specific primers were designed based on RHDV GI.1VP60 gene, and the reaction conditions were optimized to establish the RT-RPA assay, which provide a suitable method for the diagnosis and detection of RHDV GI.1.

## 2. Materials and methods

### 2.1. Ethics statement

The animal experiments were approved by the Committee on the Ethics of Animal of Institute of Poultry Science, Shandong Academy of Agricultural Sciences (permit number: 2019005), according to the guidelines of the Review of Welfare and Ethics of Laboratory Animals authorized by the Shandong Municipality Administration Office of Laboratory Animals. The animal Experiments were conducted in the Biosafety Level 2 laboratory in Shandong Academy of Agricultural Sciences, in compliance with the ARRIVE guidelines. All applicable international, national, and/or institutional guidelines for the care and use of animals were followed. Rabbits were sacrificed by well-trained operators by inhaling CO_2_ (50–60%) to protect animal welfare using animal sacrifice device.

### 2.2. RT-RPA primer's design

To establish the RT-RPA assay, the primers were designed based on the VP60 nucleotide sequence of RHDV GI.1 LW/2015 strain (Genbank accession number: KY398843): Primer: F: 5'- TGTTATGGAGGGCAAAACCCGCACAGCGCCGCAA−3'; Primer R: 5'- CGGCTGTGAATGGGTTGTTCTGTGGAGAGTGTT -3'.

### 2.3. Optimization of reaction conditions of the RT-RPA assay

The RT-RPA assay was carried out by using TwistAmp Basic kit (TWISTDX Ltd., Babraham, UK) and in a 0.2 ml reaction tube, the total reaction volume is 50 mL, including rehydration buffer 29.5 μL, Primer F (10 μM) 2.4 μL, Primer R (10 μM) 2.4 μL, template 2.0 μL, ddH2O 11.2 μL, MgAc (280 mM) 2.5 μL. Then reaction tubes were incubated at 37°C for 30 min in a metal bath, and the reaction product was observed by electrophoresis in 1.2% agarose gel and purified by axyprep DNA gel extract Kit (AXYGEN Ltd., Silicon Valley, USA). The purified amplification product was verified by sequencing (Invitrogen Trading Ltd., Shanghai, China) and analyzed using the BLAST program in National Center for Biotechnology Information website. To achieve optimal amplification results, reaction temperature and concentrations of primers were optimized. Reaction was carried out 35 to 42°C for about 30 min according to TwistAmp Basic Kit. Then, different temperature (35, 37, 39, 40, 42°C) and various primer concentrations (0.12, 0.24, 0.48 μM) were also investigated in this study, respectively.

### 2.4. Specificity and sensitivity of the RT-RPA assay

The specificity of RT-RPA assay was assessed against other common infecting viruses in rabbit including rabbit rotavirus (Z3171 strain), rabbit coronavirus (J103 strain), rabbit astrovirus (Z721 strain), Salmonella (sm1378 strain), Klebsiella pneumoniae (kp1806 strain) and Pseudomonas Aeruginosa (pa2013 strain). In addition, the liver from SPF rabbits was used as negative control.

For sensitivity analysis, infected rabbit liver tissue was grinded with PBS buffer, diluted from 10^−1^ to 10^−8^, and RNAs were extracted from those samples. The extracted RNA was then analyzed with RT-RPA method to investigate the minimum detection dose. The RT-PCR was also performed using the same diluted samples.

### 2.5. Clinical samples detection and epidemiological investigation and analysis

Seven 8-week-old SPF rabbits were purchased from Institute of animal husbandry and veterinary medicine, Shandong Academy of Agricultural Science. Five rabbits were inoculated intraperitoneally with 0.5 mL of the RHDV GI.1 NJ strain (Genbank: HM623309, provided by doctor Wang Fang of Jiangsu Academy of Agricultural Sciences) liver suspension (10^6^ LD_50_) and two rabbits were utilized as negative control with Phosphate Buffered Saline (PBS) inoculation and two rabbits were used as negative control by inoculation of Phosphate Buffered Saline (PBS). Nasal swabs were collected from infected rabbits every 12 h, and liver tissues from dead rabbits and SPF rabbits were collected for detection by RT-RPA and RT-PCR. Shandong Province is a big rabbit breeding province in China, with the output ranking among the top in the country. Therefore, 1,144 nasal swabs were randomly collected from 56 rabbit farms in Liaocheng, Linyi and Zaozhuang counties of Shandong Province.

## 3. Results

### 3.1. Establishment of RT-RPA assay

**Figure 1:**
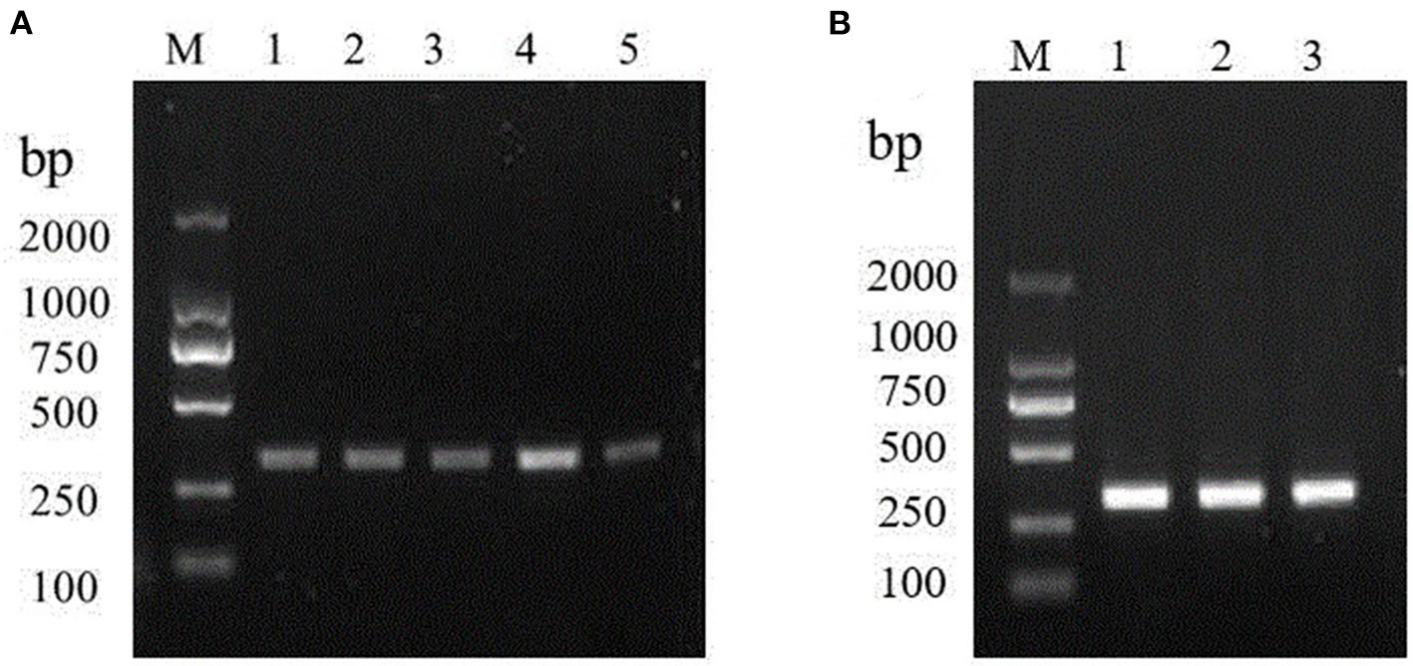
Optimizations of the RT-RPA assay components. **(A)** RT-RPA reaction temperature; M: DL2000 marker. Lanes 1–5: 35, 37, 39, 40, and 42°C, respectively. **(B)** Optimal primer concentration screening. M: DL2000 marker. Lanes 1-6: 0.12, 0.24, and 0.48 μM, respectively.

The specificity of the amplification product was confirmed and visualized by agarose gel as 317 bp bands, which was consistent with the expected fragment size. So, the primer-set could be utilized in the later assays. Initially, we assessed a series of temperature during in cubation for 30 min. Amplification signals can be detected at 35, 37, 39, 40, 42°C, among which the amplification signal was in the highest at 40°C (Figure 1A). Amplification signals could be detected at 40°C various primer concentrations 0.12, 0.24, and 0.48 μM. However, there was no significant difference in the products yielded among the 0.12, 0.24, and 0.48 μM reactions (Figure 1B).

### 3.2. Specificity of the RT-RPA assay

**Figure 2:**
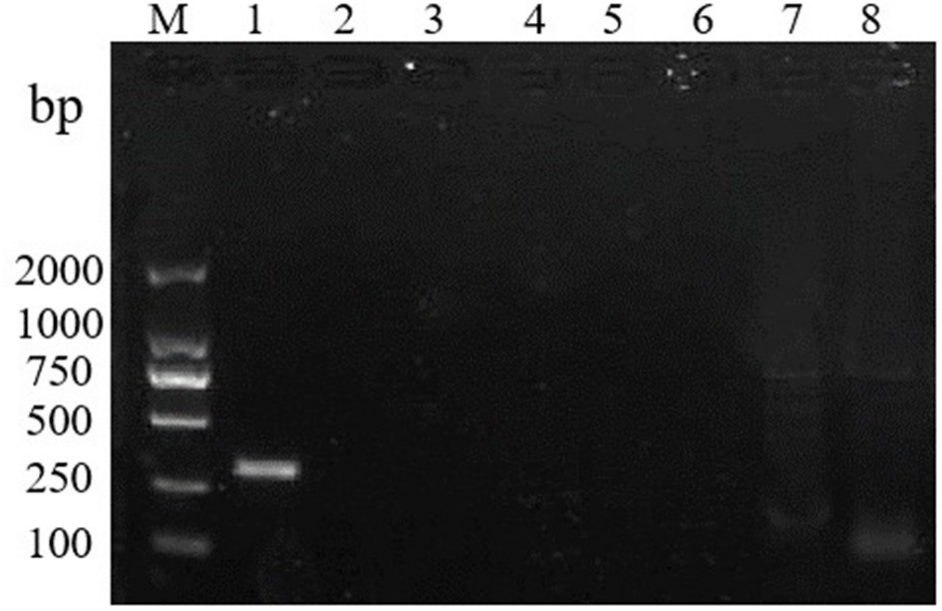
RT-RPA specificity test. M: DL2000 marker. Lanes 1–8: RHDV, rabbit rotavirus, rabbit coronavirus, rabbit astrovirus, Salmonella, Klebsiella pneumoniae, Pseudomonas Aeruginosa, and SPF rabbit liver.

To test the specificity of the RT-RPA assay, RHDV GI.1 and 6 other pathogens were detected by the assay. The results show that, only the RHDV GI.1 but no other pathogens used in this study was detected indicating that the primers showed a high specificity (Figure 2).

### 3.3. Sensitivity of the RT-RPA assay

**Figure 3:**
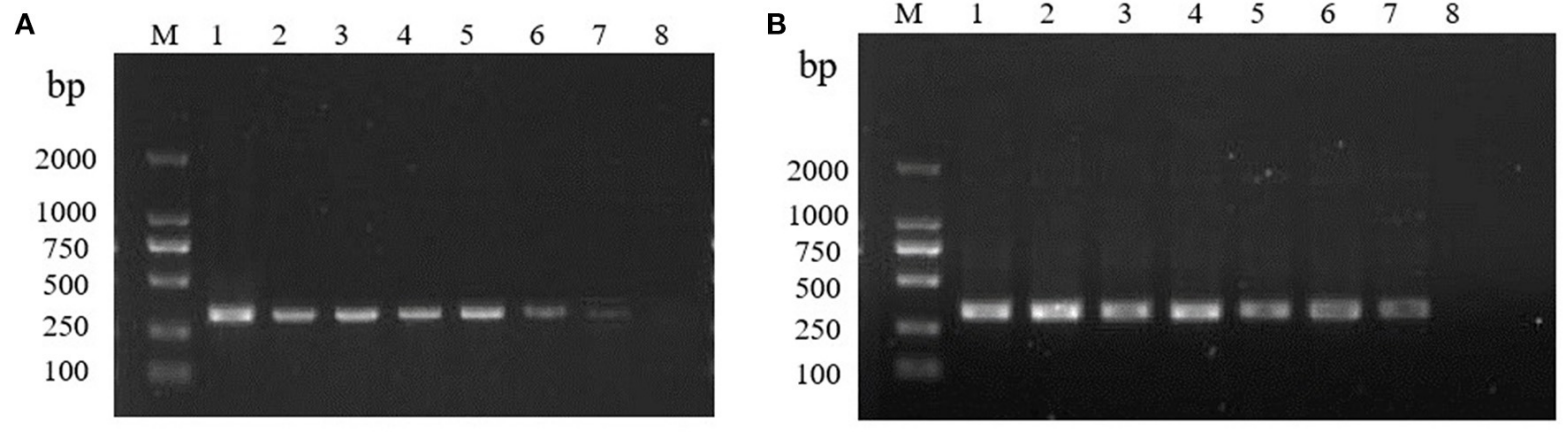
Sensitivity of the RT-RPA and RT-PCR. **(A)** Sensitivity of the RT-PCR. M: DL2000 marker. 10-fold gradient dilution of the RNA samples (10 ^−1^ −10 ^−8^, lanes 1–8). **(B)** Sensitivity of the RT-RPA. M: DL2000 marker. 10-fold gradient dilution of the RNA samples (10 ^−1^ −10 ^−8^, lanes 1–8).

The RT-RPA assay was assessed for the detection sensitivity using different concentrations of the RHDV GI.1 RNA standards under the optimized reaction system. The RT-RPA assay showed high sensitivity, with the detection limit is 0.1LD_50_ of RHDV GI.1. The results show that RT-PCR assay and RT-RPA assay had the same detection limit 0.1LD_50_ of RHDV GI.1 (Figures 3A, B).

### 3.4. Samples detection and epidemiological investigation using the RT-RPA assay

**Figure 4:**
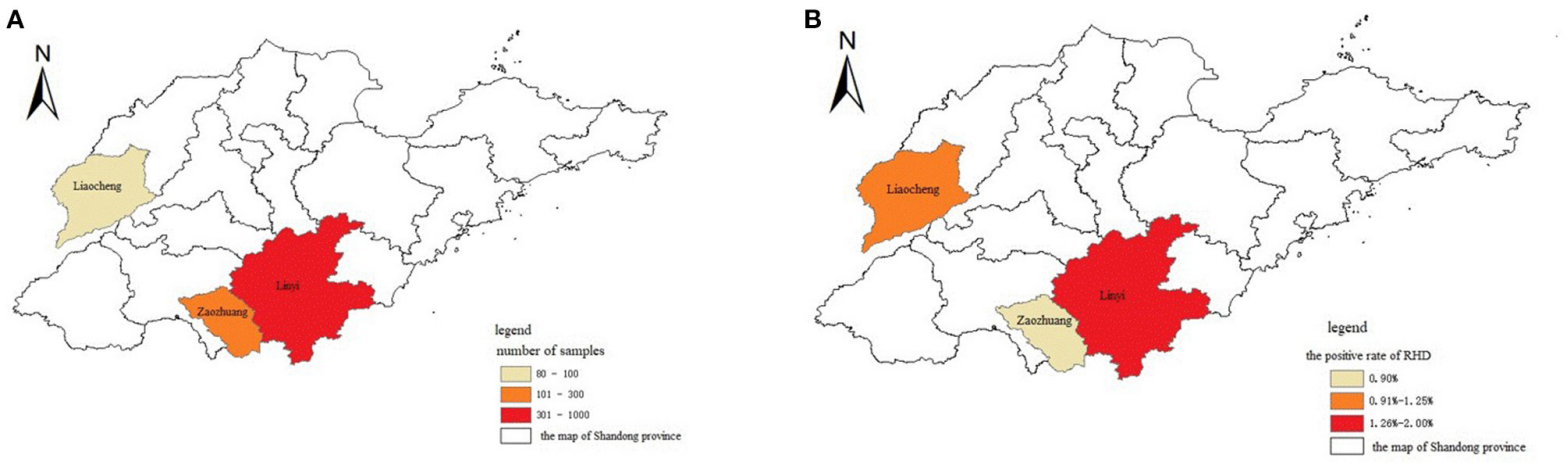
Geographic distribution and epidemic of RHDV. **(A)** The cities where the samples were collected were indicated in different colors in the map of Shandong province in China. **(B)** The cities affected with RHDV infection were indicated in different colors in the map of Shandong province in China.

Nasal swabs were collected at 12, 24, and 36 h after infection, of which 3 rabbits died at 36 h and 2 rabbits died at 48 h. The results of RT-RPA and RT-PCR showed that all nasal swabs from five rabbits infected artificially with RHDV GI.1 were detected positive 12 h post infection, the liver of dead rabbits was positive, and the liver of SPF rabbits was negative (Table 1). Furthermore, among 1,144 nasal swab samples, 80 were from Liaocheng, 854 from Linyi, and 210 from Zaozhuang (Figure 4A). The results showed that the positive rate was 0.95, 1.29, 2.50% in Zaozhuang, Linyi, Liaocheng, respectively (Figure 4B). The average positive rate is 1.58% in three area, However, there was no significant difference in RHDV GI.1 infection in different regions [the Fisher's exact test, *P* > 0.05 (*P* = 0.413)]. The established RT-RPA method was consistent with one-step RT-PCR detection, with 1.22% positive rate (14/1,144) (Table 2). There were 16.1% (9/56) rabbit farms positive for RHDV GI.1. The detection rate of positive samples was 4.26% at the age of <3 months, 1.24% at the age of 4–8 months and 0.56% at the age of 9–12 months. Most of the positive samples were found in farm with the scale of about 500 rabbits. These data indicated that although RHDV GI.1 vaccine was immunized in selected rabbit farms, field RHDV GI.1 infection still existed in some rabbits.

**Table 1:** Results of detection of artificially infected rabbit samples by RT-PCR and RT-RPA.

**Sampling frequency**	**Detection method**		**Agreement (%) (RT-RPAandRT-PCR)**
	**RT-PCR**	**RT-RPA**	
	**Positive/Number**	**Positive/Number**	
12 h	5/7	5/7	100
24 h	5/7	5/7	100
36 h	5/7	5/7	100
48 h	2/4	2/4	100

**Table 2:** Details and detecting results of the nasal swab samples.

**Sample source**	**Number of farms**	**Number of samples**	**Number of positive samples (RT-RPA)**	**Positive rate of samples (%)**	**Number of positive farms**	**Positive rate of farms (%)**	**Number of positive samples (RT-PCR)**	**Agreement (%) (RT-RPAandRT-PCR)**
Liaocheng	5	80	1	2.50	1	20	1	100
Linyi	37	854	11	1.29	6	16.22	11	100
Zaozhuang	14	210	2	0.95	2	14.29	2	100
Total	56	1,144	14	1.22	9	16.1	14	100

## 4. Discussion

RHDV GI.1 generally infects adult rabbits and has the characteristics of rapid spread, highly contagious, short incubation period, and high rates of mortality and morbidity, which brings significant economic losses to the rabbit industry. Through the observation of five rabbits with acute death caused by artificial infection, it can be seen that the disease is a kind of systemic sepsis, there are serious material metabolism disorders in the body, many important parenchymal organs show progressive lesions and bleeding, and the defense mechanism of the body collapses, so the infected rabbits die quickly after infection. Although there is a commercial vaccine for RHDV GI.1, the rabbits still have infection after immunization Therefore, it is vital for establish a method for rapid detection of RHDV GI.1.

HA test was the first routine laboratory diagnostic method used for RHD detection. RHDV GI.1 only agglutinated with human red blood cells, but not agglutinate with rabbit or other mammalian red blood cells. However, it was difficult to obtain human O-type blood in most of laboratories because of the public health conditions (11). In addition, it was found that the emergence of no agglutinated “O” type red blood cell characteristic strain may lead to missed detection and wrong detection. Through electron microscopy (12), especially immune electron microscopy (IEM), virus particles and virus proteins can be accurately identified, but it was not suitable for clinical diagnosis since it required expensive equipment and well-trained technicians.

To date, molecular assays include conventional RT-PCR, real-time fluorescent quantitative RT-PCR, etc. have been widely used in most labs. However, these methods need special equipment, and require the experimenters to spend time exploring a stable reaction system, and the reaction time is too long. Compared with the previous detection techniques, RT-RPA method has the advantages of fast, sensitive, simple operation and short-time.

Nowadays, RPA method is widely used in the detection of a variety of different fields including human and veterinary pathogenic microorganism detection, food safety and agriculture (13–15). The method not only offset the faultiness of other methods, such as low detection rate, time-consuming, and high requirements for instruments, but also improves the detection efficiency, reduces the workload, and shortens the detection time. In addition, since all RT-RPA reagents were commercially available, it was conducive to the standardization of this assay.

However, RT-RPA detection methods still have some limitations. In the study, using the LW/2015 strain as a reference, we optimized the primer sequence by aligning it to the known RHDV GI.1 sequence in GenBank. The primer is relevantly conserved for RHDV GI.1 in theory. The above tests have demonstrated that RT-RPA is a viable method for detecting RHDV GI.1. In the future, we will consider the degenerate bases of the primer for the potential mutation of the VP60 gene of newly isolated RHDV GI.1.

For this RT-RPA assay, the amplified fragments detected by agarose gel electrophoresis can be observed within 30 min. The optimal reaction conditions were determined through optimizing the concentration of the primers and reaction temperature, and the optimal reaction temperature was determined to be 40°C. Then, specificity and sensitivity tests were performed compared with normal RT-PCR assay. The results showed that the method had good specificity and had no cross-reaction with other pathogen which are often susceptible rabbits, such as E. coli, Pasteurella, Salmonella, Bordetella and SPF rabbit liver. The sensitivity of the RT-RPA method and normal RT-PCR method was the same, with the detect limit is 0.1LD_50_ of RHDV GI.1.

The most effective preventive measure for RHDV GI.1 is vaccination. Despite the vaccination, the disease is still sporadic and epidemic. To understand the prevalence of RHDV GI.1 in Shandong Province, 1,144 samples were detected with the RT-RPA method established in this study. The results showed that the established RT-RPA method was consistent with RT-PCR one-step detection, with 14 RHDV GI.1 positive samples were detected, the positive detection rate was 1.22%, which had good clinical applicability. And the veterinary epidemiological investigation and monitoring sampling calculator showed that 23 samples were collected in farm with the scale of about 500 rabbits. To provide basis for local RHD etiology monitoring and RHD prevention and control, so as to better ensure the healthy and sustainable development of rabbit industry.

In conclusion, an RT-RPA assay was established for RHDV GI.1 detection in this study. The method has the advantages of good stability, strong specificity and high sensitivity, which had a good application prospect and provided a technique tool for clinical diagnoses and epidemic disease monitoring of RHDV GI.1 in the future.

## Data availability statement

The raw data supporting the conclusions of this article will be made available by the authors, without undue reservation.

## Ethics statement

Animal experiments were approved by the Committee on the Ethics of Animal of Institute of Poultry Science, Shandong Academy of Agricultural Sciences (permit number: 2019005), according to the guidelines of the Review of Welfare and Ethics of Laboratory Animals authorized by the Shandong Municipality Administration Office of Laboratory Animals.

## Author contributions

Conceived and designed the study: BH and LZZ. Performed the study: LZZ, QYZ, YTi, and YTa. The data analysis and manuscript revision: LZZ, YXW, and BH. Wrote the manuscript: LZZ. All authors have read and approved the final manuscript.
